# An Fc-muted bispecific antibody targeting PD-L1 and 4-1BB induces antitumor immune activity in colorectal cancer without systemic toxicity

**DOI:** 10.1186/s11658-023-00461-w

**Published:** 2023-05-31

**Authors:** Lian-sheng Cheng, Min Zhu, Yan Gao, Wen-ting Liu, Wu Yin, Pengfei Zhou, Zhongliang Zhu, Liwen Niu, Xiaoli Zeng, Dayan Zhang, Qing Fang, Fengrong Wang, Qun Zhao, Yan Zhang, Guodong Shen

**Affiliations:** 1 Department of Geriatrics, The First Affiliated Hospital of University of Science and Technology of China, Gerontology Institute of Anhui Province, Division of Life Sciences and Medicine, University of Science and Technology of China, Hefei, 230001 Anhui China; 2 Hefei HankeMab Biotechnology Limited, Hefei, 230088 Anhui China; 3 Anhui Provincial Key Laboratory of Tumor Immunotherapy and Nutrition Therapy, Hefei, 230001 Anhui China; 4 Anhui Province Key Laboratory of Gene Engineering Pharmaceutical, Biomedicine Technology Innovation Center of Hefei, Anhui Anke Biotechnology (Group) Co., Ltd., Hefei, 230088 Anhui China; 5 School of Life Sciences, Division of Life Sciences and Medicine, University of Science and Technology of China, Hefei, 230026 Anhui China; 6 School of Health Service Management, Anhui Medical University, Hefei, 230032 Anhui China

**Keywords:** Colorectal cancer, Cancer immunotherapy, 4-1BB/CD137, PD-1/PD-L1, Immune checkpoint inhibitor, Antitumor immunity

## Abstract

**Background:**

Resistance to immune checkpoint inhibitor (ICI) therapy narrows the efficacy of cancer immunotherapy. Although 4-1BB is a promising drug target as a costimulatory molecule of immune cells, no 4-1BB agonist has been given clinical approval because of severe liver toxicity or limited efficacy. Therefore, a safe and efficient immunostimulatory molecule is urgently needed for cancer immunotherapy.

**Methods:**

HK010 was generated by antibody engineering, and the Fab/antigen complex structure was analyzed using crystallography. The affinity and activity of HK010 were detected by multiple in vitro bioassays, including enzyme-linked immunosorbent assay (ELISA), surface plasmon resonance (SPR), flow cytometry, and luciferase-reporter assays. Humanized mice bearing human PD-L1-expressing MC38 (MC38/hPDL1) or CT26 (CT26/hPDL1) tumor transplants were established to assess the in vivo antitumor activity of HK010. The pharmacokinetics (PK) and toxicity of HK010 were evaluated in cynomolgus monkeys.

**Results:**

HK010 was generated as an Fc-muted immunoglobulin (Ig)G4 PD-L1x4-1BB bispecific antibody (BsAb) with a distinguished Fab/antigen complex structure, and maintained a high affinity for human PD-L1 (KD: 2.27 nM) and low affinity for human 4-1BB (KD: 493 nM) to achieve potent PD-1/PD-L1 blockade and appropriate 4-1BB agonism. HK010 exhibited synergistic antitumor activity by blocking the PD-1/PD-L1 signaling pathway and stimulating the 4-1BB signaling pathway simultaneously, and being strictly dependent on the PD-L1 receptor in vitro and in vivo. In particular, when the dose was decreased to 0.3 mg/kg, HK010 still showed a strong antitumor effect in a humanized mouse model bearing MC38/hPDL1 tumors. Strikingly, HK010 treatment enhanced antitumor immunity and induced durable antigen-specific immune memory to prevent rechallenged tumor growth by recruiting CD8+ T cells and other lymphocytes into tumor tissue and activating tumor-infiltrating lymphocytes. Moreover, HK010 not only did not induce nonspecific production of proinflammatory cytokines but was also observed to be well tolerated in cynomolgus monkeys in 5 week repeated-dose (5, 15, or 50 mg/kg) and single-dose (75 or 150 mg/kg) toxicity studies.

**Conclusion:**

We generated an Fc-muted anti-PD-L1x4-1BB BsAb, HK010, with a distinguished structural interaction with PD-L1 and 4-1BB that exhibits a synergistic antitumor effect by blocking the PD-1/PD-L1 signaling pathway and stimulating the 4-1BB signaling pathway simultaneously. It is strictly dependent on the PD-L1 receptor with no systemic toxicity, which may offer a new option for cancer immunotherapy.

**Supplementary Information:**

The online version contains supplementary material available at 10.1186/s11658-023-00461-w.

## Background

Colorectal cancer (CRC) is one of the most common malignancies worldwide, and the patient survival rate remains unacceptably low [1]. Immune checkpoint inhibitor (ICI) therapy, especially for the blockade of programmed cell death 1 (PD-1)/programmed cell death ligand 1 (PD-L1) interactions, has fundamentally revolutionized the treatment of various cancers, including CRC [2–4]. However, a large proportion of cancer patients do not respond to PD-1/PD-L1 blockade therapy [5–7], highlighting the need to find new therapeutic strategies to enhance antitumor efficacy.

4-1BB, also named CD137 or tumor necrosis factor receptor superfamily member 9 (TNFRSF9), belongs to the tumor necrosis factor receptor (TNFR) superfamily and is an important costimulatory molecule expressed functionally on the surface of T and natural killer (NK) cells and other types of leukocytes, and its clustering on the cell membrane initiates downstream signaling for cellular proliferation, activation, and cytokine secretion [8–10]. Over the past decade, the potential of 4-1BB costimulation as an effective strategy for cancer immunotherapy has been extensively demonstrated in multiple studies [11–14]. Nevertheless, several 4-1BB agonistic molecules have suffered setbacks due to notable toxicity or suboptimal efficacy, such as urelumab (BMS) with dose-limiting liver toxicity and utomilumab with insufficient activity even in combination with anti-PD-1 treatment [15–18]. Recently, the 4-1BB receptor, as a compelling target to avert the exhaustion of tumor-infiltrating lymphocytes (TILs) and overcome resistance to ICI therapy, has been supported by new immunological theories and findings [19–21]. Moreover, several bispecific antibodies (BsAbs) targeting PD-1/PD-L1 and 4-1BB, and a trispecific antibody NM21-1480 against PD-L1, 4-1BB, and serum albumin have been reported to exhibit high antitumor effects with low toxicity in preclinical models, which demonstrates that it should be a promising treatment option using an effective BsAb targeting PD-1/PD-L1 and 4-1BB [22–25].

Here, we generated a fully human Fc-muted immunoglobulin (Ig)G4 BsAb HK010 targeting PD-L1 and 4-1BB to activate 4-1BB signaling strictly dependent on PD-L1, and to simultaneously block PD-1/PD-L1 signaling within the tumor microenvironment (TME). Functional studies in vitro and in humanized mouse models indicated that HK010 significantly enhanced antitumor immunity by recruiting and stimulating TILs. Moreover, HK010 exhibited a highly safe feature in cynomolgus monkeys.

## Methods

### Cell culture

Recombinant PD-1/NFAT reporter Jurkat cells (no. 79687) and TCR-activator PD-L1/CHO cells (no. 60536) were purchased from BPS Bioscience (San Diego, USA), and FcγRIIIa (158 V) Jurkat effector cells (GM-C05619) were purchased from Genomeditech (Shanghai, China). HEK-293/NFκB-Luci/4-1BB cells were genetically engineered and expressed human 4-1BB and a luciferase reporter driven by a response element sensitive to 4-1BB agonistic stimulation and cultured in Dulbecco’s modified Eagle medium (DMEM, HyClone) supplemented with 10% fetal bovine serum (FBS, Gibco), 1% penicillin/streptomycin (P/S, HyClone), 1 μg/ml puromycin (Gibco), and 800 μg/ml hygromycin B (Sangon Biotech, Shanghai). CHO-K1-hPD-L1 cells expressing full-length human PD-L1 and CHO-K1-h4-1BB cells expressing full-length human 4-1BB were generated by lentiviral transduction. CHO-K1/CD32A, CHO-K1/CD32B, and CHO-K1/CD16 cells were designed to express human FcγRIIA, FcγRIIB, and FcγIRA on the cell membrane, respectively. All of the engineered cells were cultured in DMEM/F12 (HyClone) supplemented with 10% FBS, 1% P/S, and 1 mg/ml geneticin (Gibco). Human peripheral blood mononuclear cells (PBMCs) were purchased from Sailybio Corporation (Shanghai) with informed consent from all donors and cultured in Roswell Park Memorial Institute (RPMI) 1640 medium supplemented with 10% FBS. The murine colorectal cancer cell lines MC38 (CBP60825) and CT26 (CBP61189) were obtained from Cobioer Biosciences (Nanjing, China). Cancer cell lines overexpressing human PD-L1 (MC38/hPD-L1 and CT26/hPD-L1) were generated and cultured in RPMI 1640 medium supplemented with 10% FBS, 1% P/S, and 1 mg/ml Geneticin. All the cell lines were cultured at 37 °C in a humidified incubator with 5% CO_2_.

### Protein expression and purification

The antibodies and antigens used in the study were generated by cloning DNA-encoding sequences independently into the multiple cloning sites of the mammalian expression vector pcDNA3.4 TOPO (Invitrogen), followed by expression in the Expi293F expression system (Gibco). The sequences of the anti-CD3 antibody (clone: OKT3) were obtained from IMGT/mAb-DB (http://www.imgt.org/mAb-DB). The sequences for PD-L1 or 4-1BB proteins were obtained from UniProt (human 4-1BB: Q07011; cynomolgus monkey 4-1BB: A9YYE7; mouse 4-1BB: P20334; human PD-L1: Q9NZQ7; cynomolgus monkey PD-L1: A0A2K6E3F1; mouse PD-L1: Q9EP73). Proteins were purified by protein A chromatography or Ni^2+^ chromatography (Cytiva). Size-exclusion chromatography over a Superdex 200 10/60 PG column (GE Healthcare) for the complex of antigen and Fab was implemented for further purification.

### Protein structure analysis

The protein solution of the PD-L1/HK010 Fab complex was concentrated to 10 mg/ml, and crystal growth was performed at 16 °C for 3 days using the sitting drop vapor diffusion method. The most suitable crystal was obtained from 18% (w/v) PEG4000 with 0.1 M Bis–tris pH 6.2 and protected with cryoprotectant containing 20% glycerin before freezing in liquid nitrogen. Crystal screening of the 180 μM 4-1BB/anti-4-1BB Fab complex was performed by the sitting drop vapor diffusion method with an equal volume of crystallization reagent. Diffraction quality crystals were obtained from 18% (w/v) PEG4000 with 0.1 M Tris pH 8.0 and protected with cryoprotectant containing 18% glycerin before freezing in liquid nitrogen. X-ray diffraction data of the complexes were collected at the experimental center of the University of Science and Technology of China (USTC) and Beamline 18U of the Shanghai Synchrotron Radiation Facility (SSRF). Diffraction data processing was executed with CrysAlis Pro [26] and XDS software [27]. Both of the complex structures were solved by the molecular replacement method using the program PHASER MR in CCP4 software [28]. The structures (PDB: 6 WKM, 3BIS) were used as search models to solve the PD-L1/HK010 Fab complex structure, while the structure (PDB: 4EDW) was used to solve the 4-1BB/anti-4-1BB Fab complex structure. The initial models were subsequently refined several cycles by the program Refmac5 in CCP4 and the program Refinement in PHENIX software alternately [29]. COOT software was used to manually revise the model during the refinement process [30]. The figures showing structures were prepared by using PyMOL software [31].

### Surface plasmon resonance measurement

Binding kinetics and affinities were measured by surface plasmon resonance (SPR). Tested antibodies were coupled on a Series S Sensor Chip CM5 (GE Healthcare) to 150 RU using an Amine Coupling Kit (GE Healthcare). Serially diluted antigens were then used to detect the binding affinities to tested antibodies using Biacore equipment (T200, GE Healthcare), and the results were analysed by Biacore T200 software (V3.1, GE Healthcare).

### BsAb-mediated cell binding assay

CHO-K1-hPD-L1 or CHO-K1-h4-1BB cells were incubated for 1 h with a series of dilutions of the antibodies used. After washing, the cells were incubated with fluorescein isothiocyanate (FITC)-conjugated goat anti-human Fc antibody (H10301, Invitrogen) for 30 min at 4 °C. The stained cells were analyzed on the CytoFlex system (Beckman, USA), and median fluorescent intensity (MFI) values were plotted against the concentration of primary antibody. Human IgG used as an isotype control was purchased from GenScript Biotech (Nanjing, China). To determine the cobinding cells mediated by the BsAb HK010, CHO-K1-h4-1BB cells stained with 5,6-carboxyfluorescein diacetate, succinimidyl ester (CFSE, Invitrogen) were cocultured with CHO-K1-hPD-L1 cells labeled with cell tracker deep red (C34565, Thermo Fisher) at a ratio of 1:1 with specified concentrations of tested antibodies for 1 h in a 96-well plate. The percentage of cobinding cells as double-positive cells was analyzed using the CytoFlex system.

### BsAb specificity determination

The binding of HK010 to PD-L1 or 4-1BB proteins (human or cynomolgus monkey) and B7 or TNF receptor superfamily proteins was analyzed by enzyme-linked immunosorbent assay (ELISA). B7 or TNF receptor superfamily proteins (human B7-1: B71-HM480; human B7-2: B72-HM486; human B7-H2: BH7-HM472; human B7-H4: BH7-HM174; human B7-H3: BH7-HM173; human CD40: CD4-HM140; human OX40: P43489; human CD27: CD2-HM127) were purchased from Kactus Biosystems (Shanghai). The Nunc Maxisorp plate was coated with 1 µg/ml of proteins in carbonate buffer at 4 °C overnight. After blocking with 1% bovine serum albumin (BSA, Gibco) at 37 °C for 2 h, serially diluted test antibodies were added to each well and incubated at room temperature for 2 h. The wells were washed and incubated for 1 h at 37 °C with horseradish peroxidase (HRP)-conjugated goat anti-human IgG Fc (146460, Jackson). After washing with a phosphate buffered solution containing 0.05% Tween-20 (PBST) solution three times, tetramethylbenzidine (TMB, Invitrogen) was added as a substrate, and the absorbance was detected at 450 nm by VersaMax (Molecular Devices). For the dual-antigen capture ELISA, human PD-L1-His was coated, and HK010 binding was detected using human 4-1BB and HRP-conjugated anti-mouse antibody (115-035-062, Jackson).

### Immunofluorescence assay

Human primary CD8+ T cells were isolated from PBMCs using a CD8+ T-cell isolation kit (557766, BD). The CD8+ T cells were activated with anti-CD3 (clone: OKT3) at 37 °C in a humidified incubator with 5% CO_2_ for 2 days, and then 800,000 activated CD8+ T cells were mixed at a ratio of 10:1 with HCC1954 cells, 2 μg/ml OKT3, and the test antibody HK010. The parental anti-PD-L1 and anti-PD-L1 antibodies in combination with anti-4-1BB were added and incubated at 37 °C for 1 h. The cells were fixed with 4% paraformaldehyde (PFA) prior to incubation with goat anti-human Alexa 488-labeled test antibodies with human Fc and goat anti-mouse Alexa 546-labeled anti-CD3 antibody with mouse Fc. After washing two times with PBS, the cells were transferred to a 20 mm glass bottom cell culture dish (NEST) precoated with polylysine. Images were then acquired with a 63× oil immersion objective on a Zeiss 800 confocal microscope.

### PD-1/PD-L1 blockade and 4-1BB agonist activity assay

A PD-1/PD-L1 blockade assay system (BPS Bioscience) was used according to the supplier’s instructions. Briefly, TCR activator/PD-L1-CHO cells (60536, BPS Bioscience) were seeded at a density of 35,000 cells per well into a white clear-bottom 96-well microplate in a 100 μl volume and cultured at 37 °C in a CO_2_ incubator overnight. PD-1/NFAT-reporter-Jurkat cells (60535, BPS Bioscience) were diluted to 4 × 10^5^/ml in the medium, mixed with the test antibodies at a 1:1 volume ratio and then added to the 96-well microplate in which the medium of the TCR activator PD-L1-CHO cells had been removed. After incubating at 37 °C for 6 h, 80 µl of firefly luciferase reagent (MA0519-2, Meilunbio) was added to each well. The plate was incubated for 10 min at room temperature and then read by SpectraMax (Molecular Devices).

To determine the 4-1BB agonist activity of HK010, HEK-293/NFκB-Luci/4-1BB, and PD-L1-positive/negative cell lines (HCC1954, HCC827, MDA-MB-231, HT29, CHO-K1-hPD-L1, or CHO-K1) were seeded in 96-well plates (Corning) at 1 × 10^4^ cells/well. Then, serially diluted HK010 was incubated with cocultured cells overnight in a CO_2_ incubator at 37 °C. Similarly, to investigate whether HK010 induces 4-1BB signaling activity dependent on FcγR crosslinking, HEK-293/NFκB-Luci/4-1BB cells and cells with FcγR expression (CHO-K1/CD32A, CHO-K1/CD32B, CHO-K1/CD16) were seeded in 96-well plates and incubated with HK010 overnight in a CO_2_ incubator at 37 °C. The next day, an equal volume of firefly luciferase reagent was added, and the luciferase activity was analyzed using SpectraMax. EC50 values were acquired using GraphPad Prism 8.0 software.

### Mixed lymphocyte reaction (MLR) assay

To generate immature dendritic cells (iDCs), CD14+ monocytes were purified from human PBMCs with a kit (17858, STEMCELL) and cultured in RPMI 1640 complete medium in the presence of 1000 U/ml GM-CSF (300-03-100UG, Peprotech) and 500 U/ml IL-4 (200–04-5UG, Peprotech) for 5 days and then cultured for another 2 days to generate mature dendritic cells (mDCs) in RPMI 1640 complete medium supplemented with 10 ng/ml interleukin (IL)-6 (780501, STEMCELL), 10 mg/ml IL-1β (200-0113-10UG, Peprotech), 10 ng/ml TNF-α (300-01A-10UG, Peprotech), and 1 µg/ml PGE2 (P860711, Macklin). Human primary CD4+ T cells were isolated from PBMCs using a kit (557767, BD) according to the manufacturer’s instructions, and then cocultured with mDCs at a ratio of 10:1 in a 96-well plate and serially diluted HK010 or other antibodies for 5 days. The concentration of interferon (IFN)-γ in the cell supernatant was measured by ELISA.

### CD8+ T-cell activation dependent on the PD-L1 crosslinking assay

Human primary CD8+ T cells were isolated from PBMCs using a kit (557766, BD). To test 4-1BB agonist activity, CD8+ T cells were adjusted to 3 × 10^5^ cells/ml and cocultured with CHO-K1-hPD-L1 cells or different PD-L1-positive tumor cell lines at 1.0 × 10^5^ cells/ml in a 96-well microplate containing 0.5 µg/ml OKT3 and incubated with HK010 or other antibodies for 3 days in a CO_2_ incubator at 37 °C. Specifically, CHO-K1-hPD-L1 cells were mixed with CHO-K1 cells at proportions of 4:0 (100%), 3:1 (75%), 2:2 (50%), 1:3 (25%), and 0:4 (0). After incubation, the concentration of IFN-γ in the cell supernatant was measured by ELISA.

### Cytotoxicity assay

To test the antibody-dependent cell-mediated cytotoxicity (ADCC) mediated by HK010, ADCC effector cells (FcγRIIIa Jurkat effector cells, 3 × 10^4^ cells/well) and target cells (HCC1954 or CHO-K1-h4-1BB cells, 1 × 10^4^ cells/well) were mixed with HK010 or other antibodies and incubated at 37 °C and 5% CO_2_ for 6 h. Human IgG4 protein was used as an isotype control, whereas anti-PD-L1 avelumab and wild-type anti-4-1BB (IgG1) acted as positive controls. Luminescence was detected by SpectraMax following the addition of an equal volume of firefly luciferase reagent.

For the complement-dependent cytotoxicity (CDC) assays, 5000 target cells (HCC1954, CHO-K1-h4-1BB) were premixed with serially diluted HK010 or other antibodies in a 96-well plate, followed by the addition of 10% normal human serum (NHS) and incubation at 37 °C and 5% CO_2_ for 4 h. Rituximab was used as a positive control. Luminescence was detected by SpectraMax following the addition of Cell Counting Kit-Luminescence reagent (SH684, Dojindo).

To detect the cytotoxicity of CD8+ T cells on PD-L1-expressing tumor cells, HCC1954 cells were cocultured with human CD8+ T cells isolated from PBMCs using a CD8+ T-cell isolation kit (557941, BD). After 3 days, a CCK-8 kit (CK04, Dojindo) was used to analyze cytotoxicity. Freshly isolated CD14+ monocytes from PBMCs using the CD14 Positive Selection Kit II (17858, Stemcell) were cultured in RPMI1640 + 10% FBS + 1000 U/ml GM-CSF (300-03-100UG, Peprotech) + 500 U/ml IL-4 (200-04-5UG, Peprotech) for 5 days. After incubation with 4 μM CFSE (C34554, Invitrogen), 3 × 10^4^ cells/well activated CD14+ monocytes or 1 × 10^5^ cells/well monocyte-derived mature dendritic cells (mDCs) were cultured with CD8+ T cells at a 1:1 ratio in a 96-well microplate containing 0.5 µg/ml anti-CD3 antibody (clone: OKT3). The cells were analyzed by flow cytometry after 3 days of culture.

### Cytokine release assay

PBMCs from healthy donors in RPMI-1640 medium with 10% FBS in 96-well flat-bottom plates (2 × 10^5^ cells/well) were treated with 10 µg/ml of the tested antibodies for 48 h. HK010 was compared with the isotype control IgG4, anti-PDL1, anti-4-1BB, and positive control OKT3. The levels of the cytokines IFN-γ, TNF-α, IL-10, IL-2, IL-6, IL-4, and IL-17a in the culture medium were measured by cytometric bead array assay (C60021, QuantoBio) according to the manufacturer’s instructions. Fluorescence signals were measured by the CytoFlex system.

### Mouse models

Eight-week-old human PD-1/4-1BB double knock-in (B-hPD-1/h4-1BB) C57BL/6 mice were purchased from Biocytogen Corporation (Beijing, China) and used according to the supplier’s instructions. Briefly, 2 × 10^6^ MC38/hPD-L1 cells mixed with Corning Matrigel in a 1:1 volume ratio were inoculated subcutaneously into the right flanks of B-hPD-1/h4-1BB mice. After the tumor size reached ~150 mm^3^, the tumor-bearing animals were randomized on the basis of tumor volume and body weight. Subsequently, antibodies were injected intraperitoneally into mice twice a week for up to 3 weeks. Tumor growth was monitored twice a week by measuring tumor length and width. Tumor volume was calculated according to the following equation: 0.5 × length × width × width. For the rechallenge experiment, 2 × 10^6^ MC38/hPD-L1 cells were implanted subcutaneously into the left flank 30 days after the final vaccination.

To investigate whether the therapeutic efficacy of HK010 was dose dependent, in the same tumor model, the tumor-bearing animals were randomized on the basis of tumor volume and body weight after the tumor size reached ~120 mm^3^, and then different doses of antibodies (0.3 mg/kg, 0.6 mg/kg, 2.0 mg/kg) were injected intraperitoneally into mice twice a week for up to 3 weeks. In addition, 1 × 10^6^ human PD-L1-expressing CT26/hPD-L1 or CT26/vector tumor cells were subcutaneously injected into the right flank of B-hPD-1/h4-1BB mice to establish two tumor models. When tumors reached an average volume of 70 mm^3^, the mice were randomized into the treatment and control groups. HK010 was given intravenously twice each week.

### Histopathology analysis

Multiplexed immunohistochemistry (mIHC) and hematoxylin and eosin (HE) assays were performed by staining 4-µm-thick formalin-fixed, paraffin-embedded whole tissue sections. For the mIHC method, briefly, deparaffinized slides were incubated with the primary antibodies sequentially with a TSA 5-color kit (abs50029-100T, Absinbio) for 30 min and then treated with an anti-rabbit/mouse horseradish peroxidase-conjugated (HRP) secondary antibody (A10011-60, Absinbio) for 10 min. Slides were washed in Tris-buffered saline (TBST) buffer and then transferred to preheated citrate solution (90 °C) before being heat treated using a microwave set at 20% of maximum power for 15 min. The primary antibodies and fluorescent dyes included anti-CD3 antibody (78588T, CST)/TSA 700, anti-CD4 (25229T, CST)/TSA 570, anti-CD8 (98941T, CST)/TSA 520, and anti-NK1.1 (NB100-77528SS, Novus)/TSA 650. Each slide was then treated with two drops of 4′,6-diamidino-2-phenylindole ( DAPI, D1306, Thermo Fisher), washed in distilled water, and manually cover slipped. Slides were air dried and photographed by an Aperio Versa 8 tissue imaging system (Leica). Images were analyzed using Indica Halo software.

### PK and toxicity study in cynomolgus monkeys

All cynomolgus monkey-related experiments were conducted at CTI Biotechnology Co. Ltd. (Suzhou). Both the 5 week intravenous (IV) repeated-dose and single-dose toxicity studies were performed in compliance with the principles of the National Medical Products Administration (NMPA). All cynomolgus monkey-related experiments were performed in accordance with standard operating procedures and complied with relevant ethical regulations. Briefly, in the single-dose pharmacokinetic (PK) study, cynomolgus monkeys were intravenously injected with HK010 at doses of 2.5, 5, and 10 mg/kg (three males and three females in each group). Serum samples for drug concentration were collected from all animals before dose administration and after 5 min, 2, 4, 8, 24, 48, 96, 144, 216, 312, 384, 480, 576, 648, 720, and 816 h. Serum concentrations of HK010 were determined by ELISA. In the single-dose toxicity study, cynomolgus monkeys were intravenously injected with HK010 at doses of 75 and 150 mg/kg (four males and four females in each group). In the 5 week repeated-dose toxicity study, cynomolgus monkeys were given HK010 (0, 5, 15, or 50 mg/kg) once a week via IV infusion. Each group consisted of five males and five females, including two recovery animals. Animals were monitored for clinical signs, including injection site reactions, body weight, and ophthalmoscopy. Safety and toxicity were assessed based on standard parameters.

### Statistical analysis

Statistical analysis was performed by one-way or two-way ANOVA and Dunnett’s T3 test using GraphPad Prism 8.0 (GraphPad Software). A *p*-value of < 0.05 was considered statistically significant.

## Results

### Construction and characterization of HK010

**Fig. 1:**
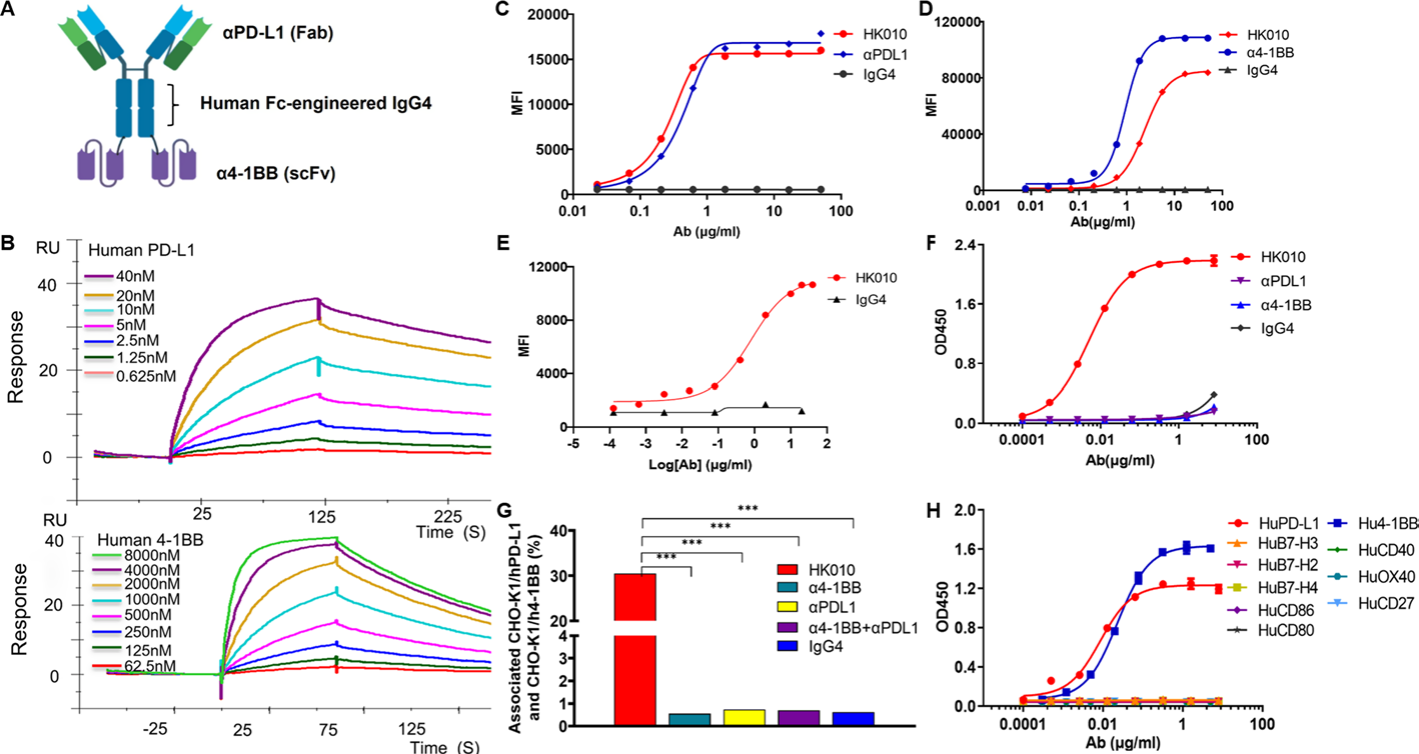
The binding activity of HK010 to both 4-1BB and PD-L1. **A** Structure overview of HK010. **B** Binding of HK010 to human 4-1BB and PD-L1 at the indicated concentrations by SPR assay. **C** Binding of HK010 to human PD-L1-expressing CHO-K1 cells measured by flow cytometry. **D** Binding of HK010 to human 4-1BB-expressing CHO-K1 cells measured by flow cytometry. **E** HK010 bound to activated CD8+ primary T cells. **F** Dual-antigen capture ELISA showed that HK010 could simultaneously bind 4-1BB and PD-L1. **G** HK010 bridged PD-L1-expressing CHO-K1 and 4-1BB-expressing CHO-K1 cells. **H** Comparison of the affinity of HK010 for members of the TNF receptor superfamily and the B7 family. The results are representative of three independent experiments. ***p < 0.001

HK010 was generated as a novel Fc-muted IgG4 PD-L1x4-1BB BsAb by fusing a single-chain variable fragment (scFv) targeting 4-1BB at the C-terminus of the heavy chain of a proprietary anti-PD-L1 IgG4 monoclonal antibody (mAb, patent CN112661854A) with three amino acid mutations (S228P, F234A, L235A) in the Fc region to mute its function (Fig. 1A). The anti-4-1BB scFv was modified from the previously generated anti-4-1BB antibody HuB6 [13] (patents CN112794906B, CN112794905A, and WO2021093753A1). HK010 was designed to maintain a high affinity for human PD-L1 (KD: 2.27 nM) and a low affinity for human 4-1BB (KD: 493 nM) to achieve strong blocking of PD-1/PD-L1 and appropriate agonism of 4-1BB, which was confirmed by SPR (Fig. 1B). The cell binding assays demonstrated that HK010 bound human PD-L1 to an equivalent level as the parental mAb (α-PDL1, Fig. 1C), and the affinity with 4-1BB was significantly reduced compared with the parental antibody (α4-1BB, Fig. 1D), which was also confirmed by ELISA (Additional file 1: Fig. S1A, B). Moreover, HK010 bound to activated CD8+ primary T cells with an EC50 of 4.09 nM, as shown by fluorescence-activated cell sorting (FACS) (Fig. 1E), and simultaneously bound to both 4-1BB and PD-L1, as shown by a dual-antigen capture ELISA (Fig. 1F). The capability of HK010 to bridge PD-L1-positive cells with 4-1BB-positive cells was further confirmed by identifying the corresponding double-positive cell population using the FACS method. In the presence of HK010, the percentage of double-positive cells was 30.5%, which was much higher than that in the IgG4 isotype control, α-PDL1, α4-1BB, or the combination of α-PDL1 with α4-1BB (< 1%) groups at the same Ab concentration (Fig. 1G, Additional file 1: Fig. S1C, D). In addition, it was confirmed that HK010 had high binding specificity to PD-L1 and 4-1BB, and showed negligible binding activities to other members of the TNF receptor superfamily and B7 family, including human OX40, CD40, CD27, CD 80, CD86, B7-H2, B7-H3, and B7-H4 (Fig. 1H).

**Fig. 2:**
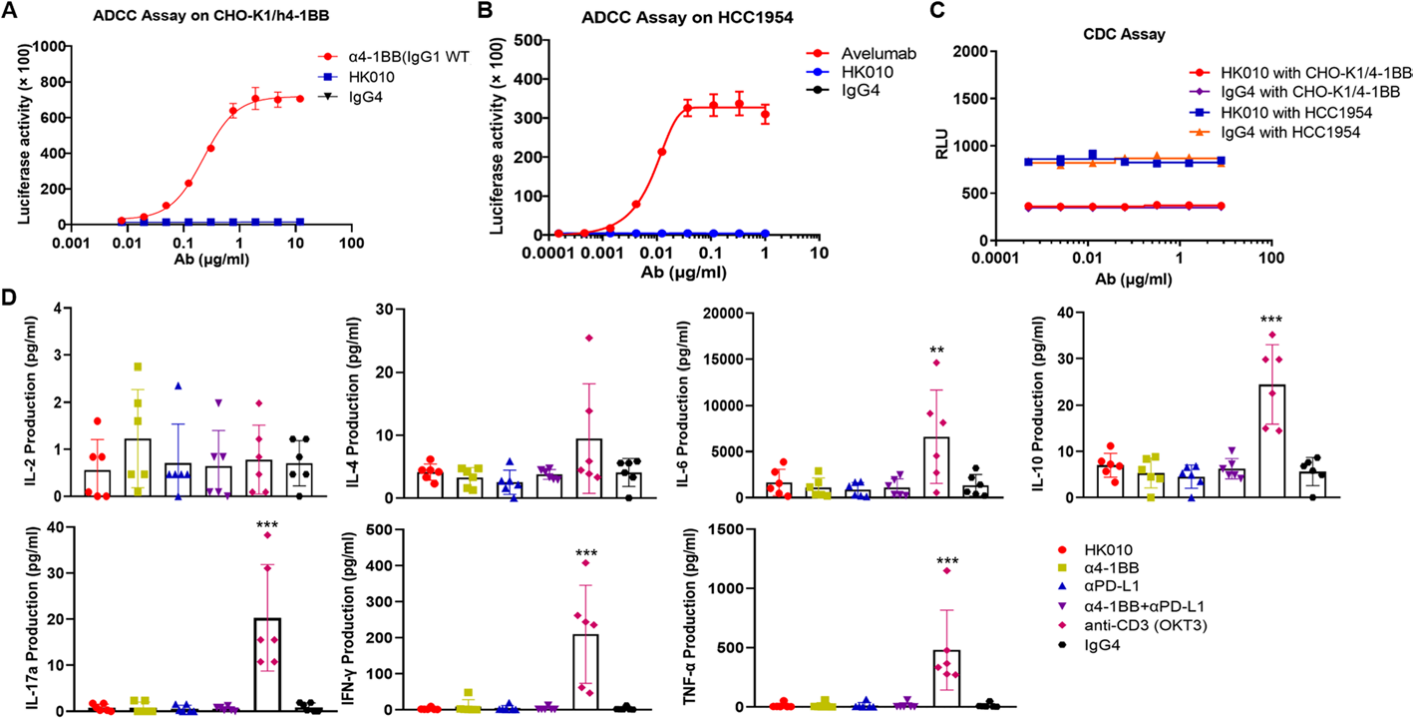
HK010 has no cytotoxicity mediated by Fc and no induced cytokine release. HK010 did not mediate ADCC against CHO-K1-h4-1BB cells (**A**) or PD-L1-expressing HCC1954 cells (**B**) according to the reporter assay. Anti-4-1BB (IgG1 WT) or anti-PD-L1 avelumab was used as the positive control to elicit ADCC activity. **C** HK010 did not mediate CDC against CHO-K1-h4-1BB cells or HCC1954 cells. **D** In vitro evaluation of HK010-induced cytokine release. A cytokine bead array (CBA) was used to evaluate the potential for cytokine release across a gradient of HK010 concentrations (*n* = 6). The values are presented as the mean ± SD from one representative of three independent experiments. **p < 0.01, ***p < 0.001

To evaluate the Fc function of HK010, cytotoxicity assays were performed, and the ADCC or CDC activity of HK010 was similar to that of the IgG4 isotype control, which demonstrated that HK010 did not mediate ADCC and CDC against PD-L1-expressing HCC1954 cells or CHO-K1-h4-1BB cells (Fig. 2A–C). Moreover, HK010 did not induce more proinflammatory cytokine (IL-2, IL-4, IL-6, IL-10, IL-17a, IFN-γ, and TNF-ɑ) release than the IgG4 isotype control in an in vitro evaluation assay (Fig. 2D). These results demonstrated that HK010 induced 4-1BB activation exclusively through PD-L1 crosslinking.

### Structural insights into the Fab and antigen complex of HK010

To obtain a deep understanding of the binding profile of our bispecific antibody HK010 toward PD-L1 and 4-1BB, we crystallized the ectodomain of PD-L1 (residues 1–136) in complex with the Fab fragment of HK010 and the ectodomain of 4-1BB (residues 24–186) in complex with the Fab of HK010. The two complex structures were both determined to 2.3 Å resolution, and the statistics for data collection, processing and structure refinement are listed in Additional file 1: Table S1.

**Fig. 3:**
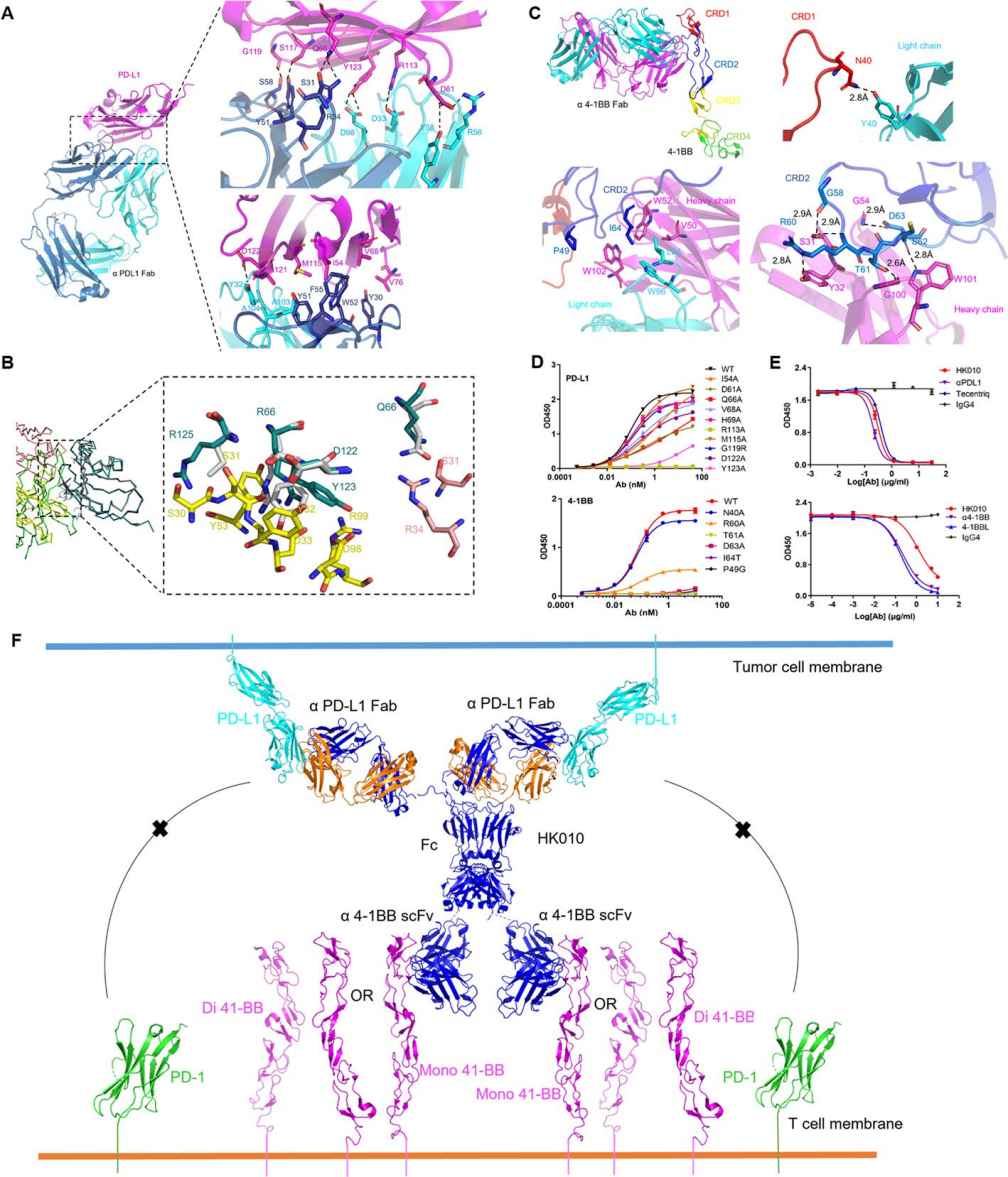
Structure diagram of the HK010 interaction with PD-L1 and 4-1BB. **A** The interface between Fab and PD-L1. Heavy chain (cyan), light chain (dark blue), PD-L1 (magenta). **B** Superposition comparison of the structures of the Fab/PD-L1 and PD-1/PD-L1 complexes in one asymmetric unit (PDB: 4ZQK). Only amino acids on PD-L1 and Fab with hydrogen bond interactions are displayed. Fab (heavy chain: yellow; light chain: pink), PD-1 (bright green), PD-L1 (combined with PD-1 status: silver, binding Fab status: dark green), and the overlapping amino acids on PD-L1 binding with Fab and PD-1 are shown in the ribbon. **C** The interface of the Fab/4-1BB complex. The 4-1BB CRDs ranging from one to four are highlighted in red, blue, yellow, and magenta, respectively. The heavy chain and light chain of anti-4-1BB Fab are shown in magenta and cyan, respectively. The interface between anti-4-1BB and 4-1BB highlighted the interactions of hydrogen bonds (dashed line) and hydrophobic interactions (lower right). **D** The affinity assay for the amino acid mutations of 4-1BB and PD-L1 in HK010. **E** ELISAs for HK010 blocking the binding of PD-L1 to PD-1 and 4-1BB ligand to 4-1BB. **F** The working model of the HK010 interaction with PD-L1 and 4-1BB. The values are presented as the mean ± SD from one representative of three independent experiments

According to the analysis of the binding interface through proteins, interfaces, structures, and assemblies (PISA) [32], HK010 has an interaction area of 982.9 Å^2^ between the anti-PD-L1 Fab and PD-L1, and the anti-PD-L1 Fab contains contact residues with PD-L1 through a number of salt bridges, hydrogen bonds, and hydrophobic interactions. Residues D53 and R76 of anti-PD-L1 Fab formed salt bridges with residues R113 and D61 of PD-L1, respectively. The anti-PD-L1 Fab also forms hydrogen bonds with residues D61, E66, R113, 117, D122, Y123, and R125 of PD-L1 (Fig. 3A). Additionally, mutations of PD-L1 at amino acids I54, V68, V76, and M115 led to a significant reduction in the affinity, consistent with the partial epitope on the face of PD-L1. Compared with the reported structure of the human PD-1/PD-L1 complex (PDB: 4ZQK), anti-PD-L1 Fab binds PD-L1 at the PD-1 competitive epitope, containing residues Q66, R113, D122, Y123, and R125 (Fig. 3B). The results demonstrated that anti-4-1BB Fab binds along the entire length of cysteine-rich domain (CRD) 2 and part of CRD1 of 4-1BB. In detail, the interface is primarily mediated by hydrogen bonds formed by the side chains of residues N40, R60, T61, and D63 of 4-1BB interacting with residues S31, Y32, Y49, and W101 of α4-1BB Fab and the main chains of residues G58 and C62 of 4-1BB interacting with residues G54 and G100 of α4-1BB Fab. In addition, hydrophobic interactions also engage in interface formation, involving residues P49 and I64 of 4-1BB (Fig. 3C).

The affinity assay of structured-based mutants in the interface was further performed. The affinity almost disappeared by mutations of PD-L1 at residues R113 and Y123, and mutations at residues D61, E66, and D122 led to a significant reduction in the affinity relative to wild-type PD-L1, which indicated that hydrogen bonds play a vital role in the formation of the complex. The anti-4-1BB Fab bound 4-1BB in a dose-dependent manner with an EC50 of 0.0706 nmol/l. In contrast, the affinity almost disappeared by mutations of 4-1BB at amino acids T61A and I64T, which indicated that both hydrogen bonds and hydrophobic interactions play a vital role in the formation of the complex. Meanwhile, mutations of 4-1BB at amino acids R60A, D63A, and P49G led to a significant reduction in the affinity, consistent with the epitope in CRD1-2 on the face of 4-1BB (Fig. 3D). Blocking ELISA revealed that HK010 blocked the binding of PD-L1 to PD-1, confirming that HK010 was a PD-1/PD-L1 blocking antibody (Fig. 3E). From the recently reported structure of the human 4-1BB/4-1BBL complex (PDB: 6BWV), we discovered that the epitope of anti-4-1BB Fab overlaps with the 4-1BBL binding site, indicating that HK010 has ligand-blocking properties, which was consistent with the results of the competitive ELISA (Fig. 3E).

Through the analysis of the binding mode of each arm of HK010 to PD-L1 and 4-1BB, we proposed a structural model of HK010 enhancing 4-1BB stimulation activity dependent on PD-L1 crosslinking (Fig. 3F). In the model, the anti-PD-L1 arm of HK010 blocks the interaction of PD-1 and PD-L1, maintaining the normal immune activity of T cells. Furthermore, the anti-4-1BB scFv combined with anti-PD-L1 Ab is able to induce 4-1BB clustering and thus initiate its downstream signaling.

### HK010 blocks the PD-1/PD-L1 interaction and selectively enhances the 4-1BB signaling activity of CD8+ T cells in a PD-L1-dependent manner

**Fig. 4:**
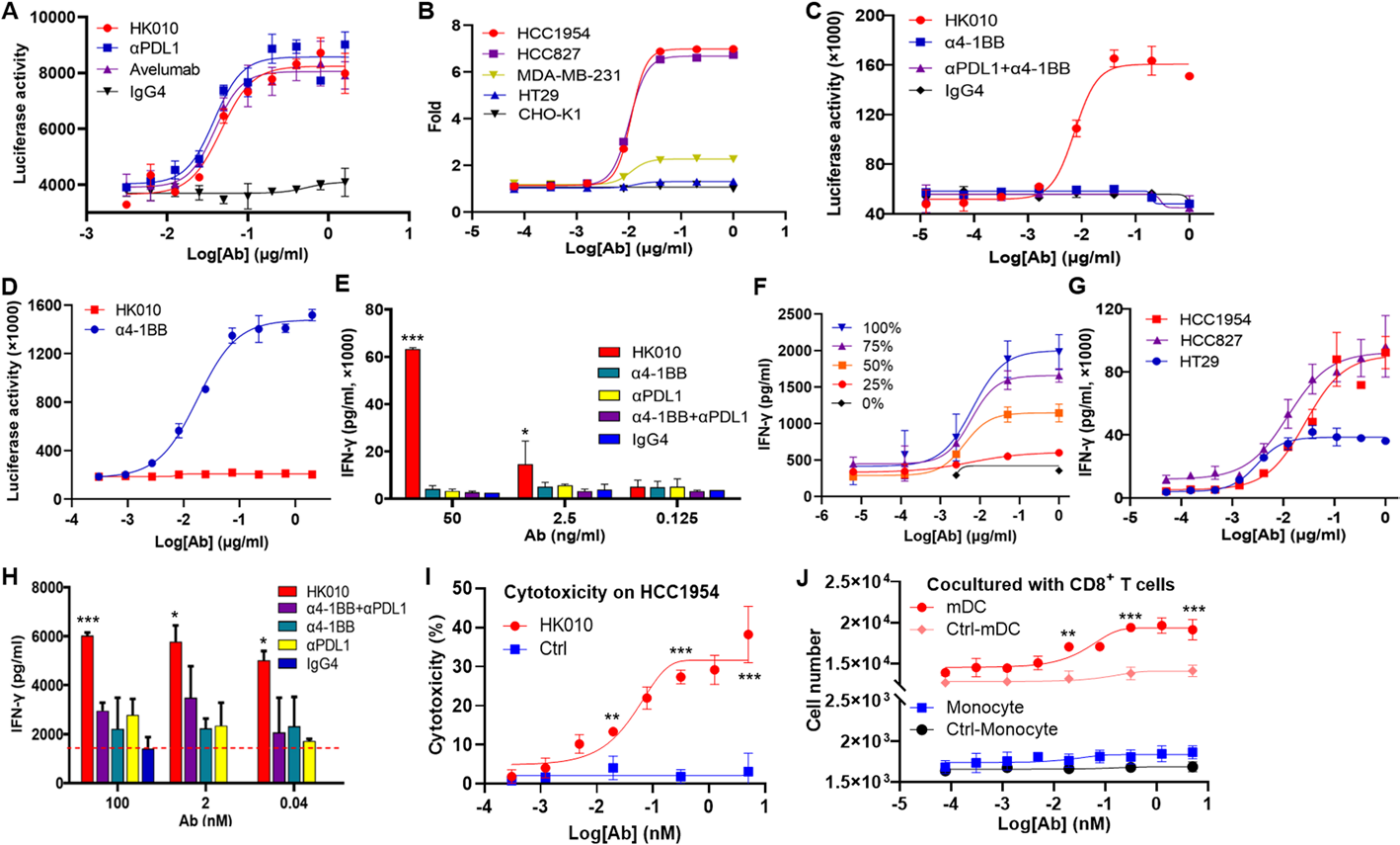
HK010 selectively enhances the 4-1BB signaling activity of CD8+ T cells in a PD-L1-dependent manner. **A** PD-1/PD-L1 blocking activities of HK010 and control antibodies were determined using the luciferase reporter assay. A human IgG4 protein was used as a negative control, whereas the avelumab and anti-PD-L1 antibodies acted as positive controls. **B** The 4-1BB signaling activities of HK010 on HCC1954, HCC827, MDA-MB-231, and HT29 cells were compared with CHO-K1 using the HEK-293/NFκB-Luci/4-1BB reporter assay. **C**, **D** The 4-1BB agonist activities of HK010 and control antibodies were determined using the luciferase reporter assay. HEK-293/NFκB-Luci/4-1BB reporter cells were cocultured with HCC1954 cells (**C**) or cells with FcγR expression (**D**). **E** The IFN-γ secretion levels of human primary CD8+ T cells cocultured with HCC1954 cells in the HK010 and control antibody treatment groups were compared. **F** The IFN-γ secretion levels of human primary CD8+ T cells treated with HK010 and cocultured with the indicated ratios of PD-L1-expressing CHO-K1 cells were compared. **G** The IFN-γ secretion levels of human primary CD8+ T cells treated with HK010 and cocultured with different tumor cells were compared. **H** The IFN-γ secretion induced by HK010 was detected in the MLR assay. The red dashed line indicates the background value of the IgG4 isotype control. **I** The cytotoxicity of HK010 on HCC1954 cancer cells was determined by flow cytometry. **J** The cytotoxicities of HK010 on human monocytes and mDCs were determined by flow cytometry. The values are presented as the mean ± SD from one representative of three independent experiments. *p < 0.05, **p < 0.01, ***p < 0.001

A PD-1/PD-L1 blockade bioassay system may be used for the in vitro blocking activity of test molecules on the PD-L1/PD-1 axis [33]. Our results showed that HK010 effectively blocked the PD-1/PD-L1 interaction, and its activity was equivalent to that of the positive control anti-PD-L1 parental antibody or avelumab (EC50 of HK010 is 0.05 μg/ml and those of positive controls are both 0.04 μg/ml. Fig. 4A), which demonstrated that the PD-L1 arm of HK010 could function as an immune checkpoint inhibitor.

To evaluate the 4-1BB agonist activity of HK010, the HEK-293/NFκB-Luc/4-1BB-cell line was constructed, and its luciferase activity regulated by NFκB reflected the activation level of the 4-1BB signaling pathway. First, the PD-L1 expression levels of the test cell lines were determined, and the expression in descending order was HCC1954, HCC827, MDA-MB-231, HT29, and CHO-K1, in which HCC1954, HCC827, and MDA-MB-231 should belong to PD-L1-positive tumor cell lines (Additional file 1: Table S2). Second, all the cell lines were cocultured with HEK-293/NFκB-Luci/4-1BB cells and various concentrations of HK010 (10^–5^–1 μg/ml). As expected, HK010 induced a PD-L1 expression-dependent increase in NFκB signaling activity in the positive tumor cell lines, whereas there was no significant change in the PD-L1-negative cell lines, which demonstrated that the 4-1BB signal-stimulating activity of HK010 correlated with the PD-L1 expression level (Fig. 4B). Third, when the cocultured HCC1954 and HEK-293/NFκB-Luci/4-1BB cells were treated with the test antibodies, only HK010 significantly stimulated 4-1BB signaling, whereas either anti-4-1BB Ab alone or in combination with anti-PD-L1 Ab did not induce luciferase activity or the IgG4 isotype (Fig. 4C). Fourth, HK010 did not induce 4-1BB signaling in the presence of cells expressing FcγR (FcγRIIA, FcγRIIB, and FcγIRA). In contrast, the anti-4-1BB parental Ab HuB6 could mediate FcγR-dependent 4-1BB activation, consistent with a previous report [13]. The data indicated that HK010 should not activate 4-1BB signaling by FcγR crosslinking (Fig. 4D).

To further confirm that HK010 activates 4-1BB signaling in a strictly PD-L1-dependent manner, human primary CD8+ T cells were cocultured with PD-L1-positive HCC1954 cells and treated with HK010 or other control antibodies. Notably, HK010 significantly increased IFN-γ secretion in the medium compared with anti-PD-L1 Ab, anti-4-1BB Ab, or their combination when the concentration was more than 2.5 ng/ml (Fig. 4E). Moreover, we found that HK010 increased IFN-γ secretion in a dose-dependent manner and was dependent on the expression of PD-L1 molecules by adding different proportions of CHO-K1-hPD-L1 cells and tumor cells with different PD-L1 expression levels (Fig. 4F, G).

PD-L1 is also expressed on antigen-presenting cells (APCs), including macrophages, dendritic cells (DCs), and monocytes, in addition to tumor cells in the tumor microenvironment [34]. To assess the ability of HK010 to stimulate CD4+ T cells, a mixed lymphocyte reaction (MLR) assay of CD4+ T cells cocultured with mDCs was used, and the results showed that HK010 induced remarkable activation of CD4+ T cells, as measured by an increase in IFNγ secretion even at a very low concentration (0.04 nmol/l). However, the combination of anti-4-1BB and anti-PD-L1 Abs only resulted in modest T-cell costimulation, similar to the single treatment of anti-PD-L1 or anti-4-1BB Ab without crosslinker, which indicated that the BsAb format should have an enhanced antitumor effect (Fig. 4H).

To test the possibility of killing PD-L1-expressing DCs and monocytes, human PD-L1-expressing cancer cells, DCs, and monocytes were cocultured with human CD8+ T cells and treated with HK010. Our results showed that HK010 activated CD8+ T cells and resulted in the destruction of HCC1954 cells when the Ab concentration was approximately 0.02 nmol/l or more (Fig. 4I, *p* < 0.01). However, HK010 treatment did not obviously change the number of human monocytes and even promoted the proliferation of mDCs when the Ab concentration was not less than 0.02 nmol/l (Fig. 4J, *p* < 0.01), in accordance with previous reports [35, 36].

### HK010 shows potent antitumor efficacy and immune memory in vivo

**Fig. 5:**
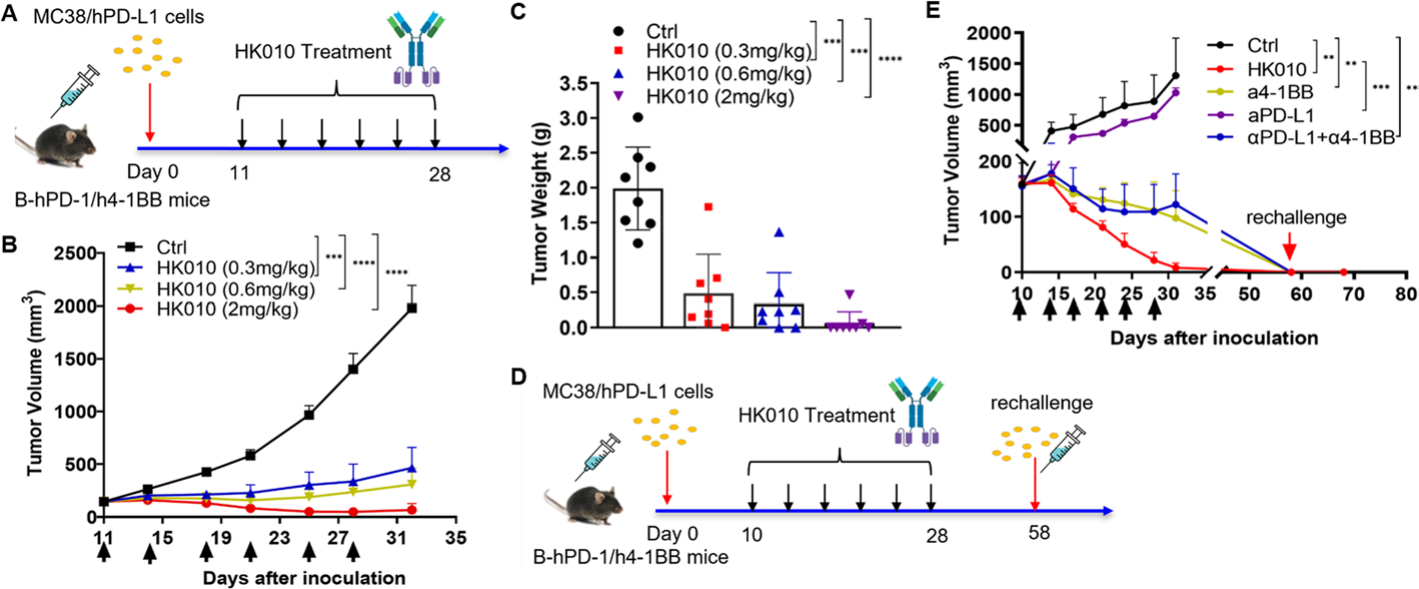
HK010 shows strong antitumor activity in vivo. **A** Schematic diagram of HK010 treatment of B-hPD-1/h4-1BB mice bearing MC38/hPD-L1 transplants. When the mean tumor size reached approximately 100 mm^3^, mice were randomized into groups of eight animals per group. Treatment with HK010 six times (indicated by vertical arrows). The changes in tumor volume (**B**) and tumor weight (**C**) of the mice treated with HK010. The values are presented as the mean ± SEM. **D** Schematic diagram of the MC38/hPD-L1 cell rechallenge experiment after HK010 treatment of B-hPD-1/h4-1BB mice bearing MC38/hPD-L1 transplants. When the mean tumor size reached approximately 150 mm^3^, mice were randomized into groups of five animals per group. Antibody treatment using HK010 or αPD-L1 plus α4-1BB was performed six times (indicated by vertical arrows), and the changes in tumor volume were compared (**E**). The values are presented as the mean ± SD from one representative of three independent experiments. **p < 0.01, ***p < 0.001

To investigate the antitumor effect of HK010 in vivo, a B-hPD-1/h4-1BB mouse model bearing MC38/hPD-L1 tumors was established and given IV HK010 according to the schematic diagram (Fig. 5A). First, we investigated whether the therapeutic efficacy of HK010 was dose dependent, and the changes in tumor volumes showed that HK010 had clear dose-dependent antitumor efficacy, with tumor growth inhibition (TGI) rates of 77.94%, 87.34%, and 96.68% at 0.3, 0.6, and 2 mg/kg, respectively (Fig. 5B). By comparing the tumor weights of all the groups, HK010 still had a marked dose–effect response (Fig. 5C). Moreover, complete tumor subsidence occurred in all three treatment groups, and the ratio was 6/8 in the 2 mg/kg HK010 group at the end of the experiment (Additional file 1: Fig. S2). Next, the antitumor efficacy and immune memory capacity between the 2 mg/kg dosage of HK010 and control antibodies were compared according to the experimental procedure (Fig. 5D), modified from a previous study [37]. There was a significant reduction in tumor volume in the HK010, anti-4-1BB HuB6, and combination anti-4-1BB and anti-PD-L1 antibody groups (*p* = 0.0023, 0.0038, and 0.0044, respectively) at the end of antibody administration compared with the saline control. Notably, HK010 induced complete tumor regression on day 35, whereas HuB6 and the two parental combinations induced complete tumor regression until day 58. Importantly, all the HK010-cured mice were protected against tumor rechallenge, indicating that HK010 could induce durable antigen-specific immunological memory (Fig. 5E). Taken together, these data indicated that HK010 induced notable antitumor activity in vivo, which seemed to be superior to a PD-L1 antagonist or 4-1BB agonist and their combination.

In addition, the effect of HK010 was compared on human PD-L1-expressing CT26 (CT26/hPD-L1) and CT26/vector tumors in Balb/c-hPD1/h4-1BB models. We found that HK010 had a significant tumor-inhibitory effect on CT26/hPD-L1 tumors. However, no obvious efficacy was observed in CT26/vector tumors treated with HK010 (Additional file 1: Fig. S3). These data confirmed that HK010 exerted an antitumor function in a strictly PD-L1-dependent manner.

### HK010 targets T cells to tumor cells

**Fig. 6:**
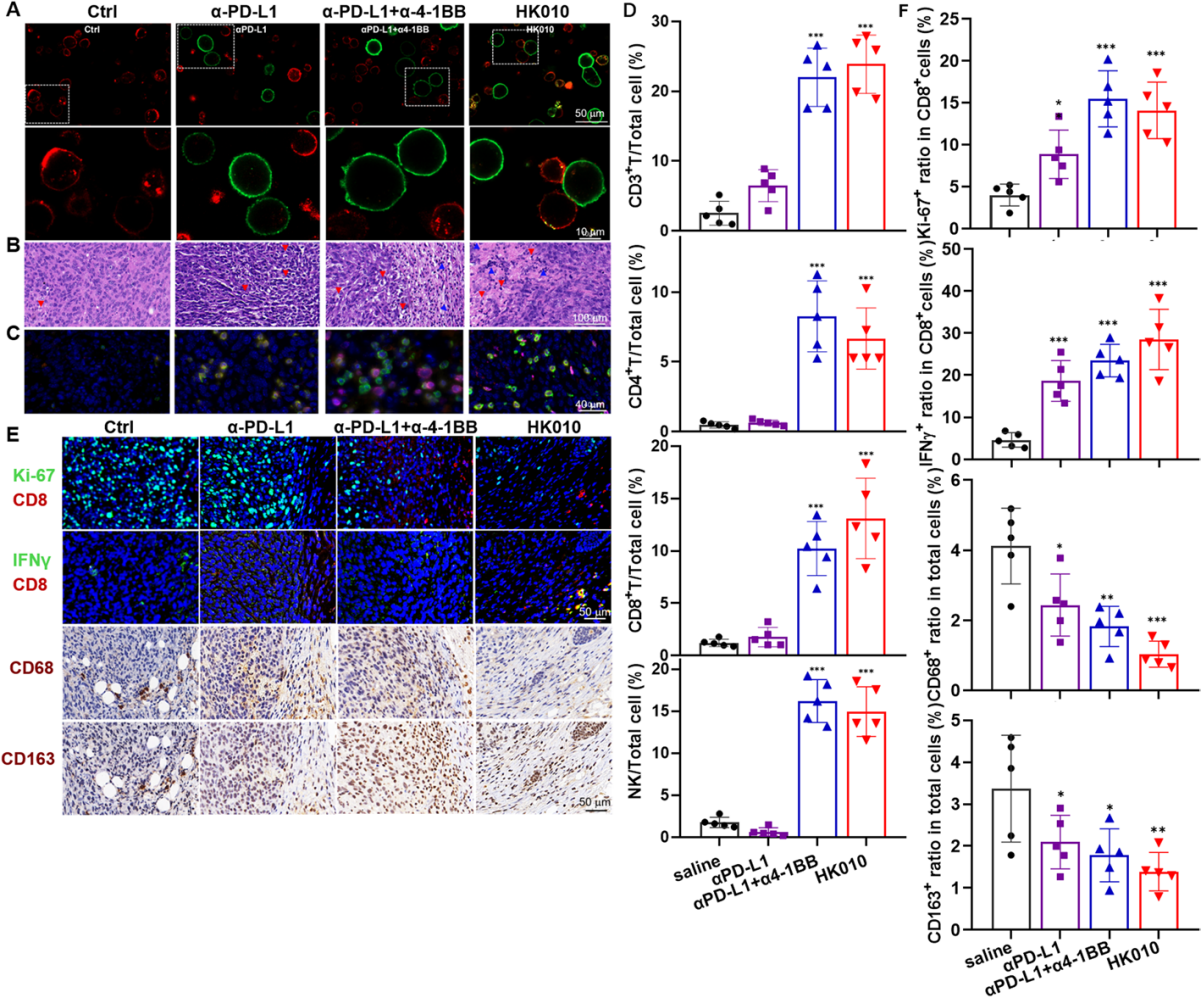
HK010 increases the abundance of T cells and NK cells in tumors and antitumor efficacy. **A** Confocal images showing the capacity of HK010 or control antibodies to bridge activated CD8+ T cells and tumor cells. CD8+ T cells were stained red using anti-CD3 antibody, and HCC1954 cells were stained green using HK010 or control antibodies and secondary antibody. Data are representative of two repeat experiments. αPD-L1 and the combination of αPD-L1 and α4-1BB failed to induce synapse formation with T cells. **B** Hematoxylin and eosin (HE) staining and **C** multiplexed immunohistochemistry (mIHC) staining for tumor tissue treated with HK010 or control antibodies. Red and blue arrows mark tumor cell apoptosis and tumor-infiltrating lymphocytes, respectively. **D** The percentages of tumor-infiltrating CD3+ T, CD8+ T, CD4+ T, and NK cells in the total cells in (**C**) are shown. **E** The expression levels of Ki-67, IFNγ, CD8, CD68, and CD163 were detected in tumor tissues treated with HK010. **F** The percentages of tumor-infiltrating Ki-67^+^ and IFNγ^+^ cells in CD8 T and CD68^+^ and CD163^+^ cells in the total cells in (**E**) are shown. The values are presented as the mean ± SD from one representative of three independent experiments. *p < 0.05, **p < 0.01, ***p < 0.001

To explore the antitumor mechanism of HK010, we first performed a confocal imaging assay to observe the ability of HK010 to bridge human activated CD8+ T cells cocultured with HCC1954 cells. In the presence of HK010, the binding cell percentage of CD8+ T cells and tumor cells markedly increased compared with the anti-PD-L1 antibody and combination with anti-4-1BB HuB6 (Fig. 6A). Next, the in vivo capacity of HK010 to target T cells to tumor cells was assessed by histopathology for MC38/hPD-L1 tumors, as shown in Fig. 5E. HE staining indicated that HK010 could induce lymphocytes to infiltrate into tumor tissue and resulted in tumor apoptosis and necrosis (Fig. 6B). Moreover, mIHC results confirmed that HK010 increased the number and percentage of TILs, including CD4+ and CD8+ T cells and NK cells, in the tumor tissue compared with the saline control and anti-PD-L1 mAb (*p* < 0.001), thereby rescuing the exhausted TILs and exerting potent antitumor immunity in vivo (Fig. 6C, D).

To further explore the activating phenotypes of tumor-infiltrating T cells induced by HK010, we detected the expression of Ki-67, IFNγ, and CD8 in tumor tissues according to a previous study [38]. The results showed that anti-PD-L1 Ab increased both Ki-67-positive CD8 T cells and IFNγ-positive CD8 T cells (*p* < 0.05), and HK010 exhibited a better effect than anti-PD-L1 Ab, similar to the combination of α-PD-L1 with α-4-1BB (Fig. 6E, F, *p* < 0.05). Meanwhile, it is well known that tumor-associated macrophage (TAM, CD68+ or CD163+) infiltration in tumors is associated with poor prognosis in cancer patients [39]. To observe the effect of HK010 on TAMs, we detected the expression of CD68 and CD163 in tumor tissues. Anti-PD-L1 Ab treatment reduced both CD68+ and CD163+ macrophages (*p* < 0.05), and HK010 had significantly higher efficacy than anti-PD-L1 Ab (Fig. 6E, F, *p* < 0.05).

### HK010 is a well-tolerated molecule in cynomolgus monkeys

**Fig. 7:**
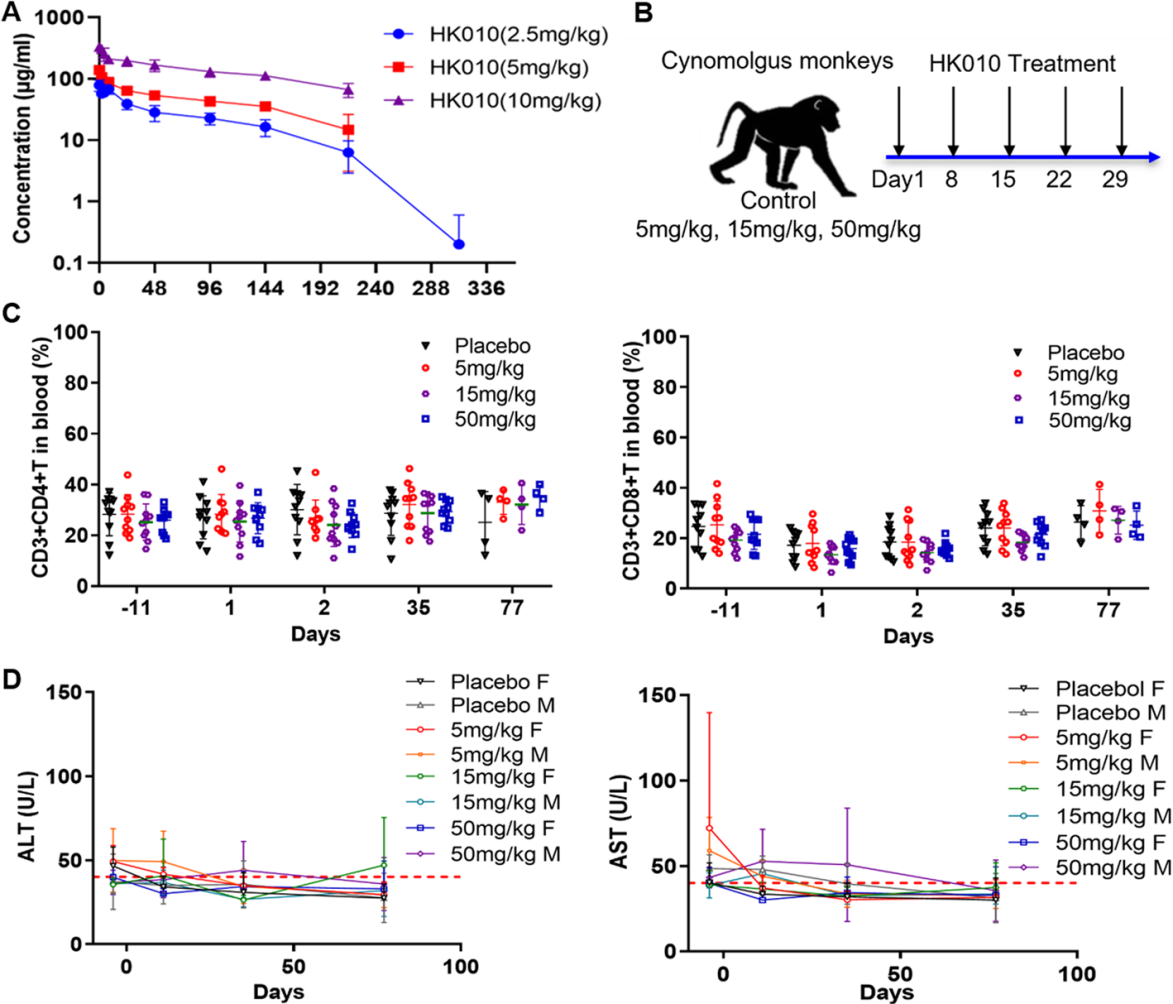
HK010 is well tolerated in cynomolgus monkeys. **A** In the single-dose PK study, HK010 displayed a dose-dependent increase in *C*_max_ and *AUC*_last_ within the dose range of 2.5–10 mg/kg. **B** Schematic diagram for the 5 week repeated-dose toxicity study. Cynomolgus monkeys were given IV HK010 treatment (0, 5, 15, and 50 mg/kg) once a week. **C** No abnormal change in the CD3+ T lymphocyte percentage in the repeated-dose toxicity study. **D** No abnormal change in blood ALT/AST levels was observed in the repeated-dose toxicity study. *M* male; *F* female. Each group had four male and four female cynomolgus monkeys. The values are presented as the mean ± SD

To evaluate safety, the cross-reactivity of HK010 in human, monkey, mouse, and rat species was first determined. HK010 had comparable affinity to the 4-1BB or PD-L1 protein of humans and cynomolgus monkeys, whereas HK010 was unable to bind to the protein from mice and rats (Additional file 1: Fig. S3). Then, the PK and safety profile of HK010 were characterized in cynomolgus monkeys by intravenous administration (Fig. 7). In the single-dose PK study, HK010 displayed a clear dose-dependent increase in the plasma concentration for doses ranging from 2.5 to 10 mg/kg (Fig. 7A). Notably, HK010 was rapidly cleared after day 9 (216 h) in the medium-/high-dose groups and approximately day 13 (312 h) in the low-dose group, which was consistent with the generation time of the anti-drug antibody (ADA) in the animal. Moreover, by a single-dose toxicity study (75 and 150 mg/kg), the maximum tolerated dose (MTD) of HK010 was determined to be 150 mg/kg in cynomolgus monkeys (four males and four females), with no observed adverse reactions, including treatment-related signs, changes in body weight, food consumption, or hematology index (data not shown).

Next, multiple-dose toxicokinetics (TK) were observed in a 5 week repeated-dose toxicity study with weekly intravenous injections of HK010 at 5, 15, and 50 mg/kg (Fig. 7B). The results showed that the area under the serum drug concentration–time curve to the last measurable serum concentration (*AUC*_last_) and maximum drug concentration observed in the serum (*C*_max_) increased in a dose-proportional relationship, and no significant sex difference was observed on day 1. However, the BsAb concentration was decreased, and there was no dose-proportional relationship between *AUC*_last_ and *C*_max_ after five doses (day 29) in the low-/medium-dose groups, which was presumably related to ADA (Table 1). Moreover, no drug-related abnormalities or changes in the hematology index, including lymphocytes and alanine transaminase/aspartate transaminase (ALT/AST) levels, were observed (Fig. 7C, D), suggesting that HK010 should have high safety in cynomolgus monkeys.

**Table 1:** Main pharmacokinetic parameters of HK010 by five intravenous injections (mean ± SD, *n* = 10)

Time	Dose (mg/kg)	*AUC*_last_ (h μg/ml)	*C*_max_ (μg/ml)	*T*_max_ (h)
D1	5	9176.1 ± 1426.5	132.4 ± 12.7	0.083–2
	15	31,452.1 ± 3108.4	381.5 ± 53.2	0.083–2
	50	105,464.1 ± 10,710.5	1203.6 ± 187.5	0.083–24
D29	5	15.0 ± 2.6	13.5 ± 1.6	0.083
	15	10,152.1 ± 14,821.3	212.3 ± 199.3	0.083–2
	50	113,820.4 ± 86,091.3	1371.8 ± 439.7	0.083–24

*AUC*_*last*_ area under the serum drug concentration–time curve to the last measurable serum concentration; *C*_*max*_ maximum drug concentration observed in the serum; *T*_*max*_ time of the first occurrence of *C*_max_

## Discussion

**Fig. 8:**
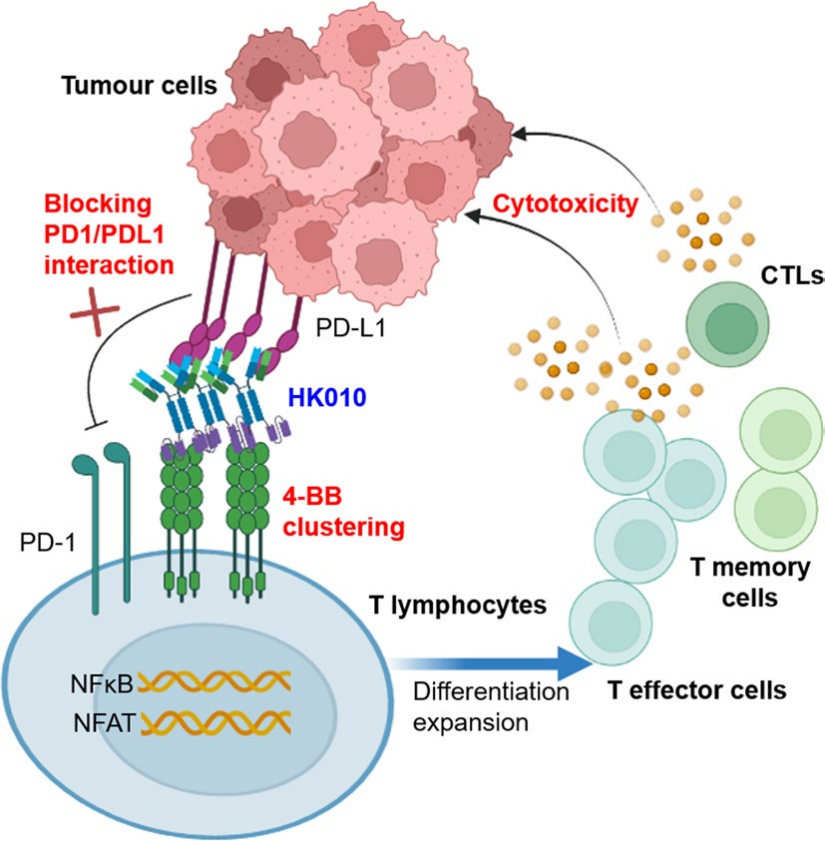
Schematic illustration of the mechanism of action of HK010. HK010 simultaneously binds to PD-L1 expressed on tumor cells and 4-1BB expressed on T lymphocytes and results in the blockage of the PD-1/PD-L1 interaction and clustering of 4-1BB molecules on the surface of T lymphocytes, which induces the activation of the NF-kB and NFAT signaling pathways and the differentiation expansion of T effector cells, T memory cells, and cytotoxic T lymphocytes (CTLs), and thus effectively and specifically promotes the death of tumor cells

**A** 4-1BB agonist triggers receptor clustering and results in downstream signaling cascades that enhance T-cell function, including cell proliferation, activation, cytokine production, and immune memory formation [40–42]. Unfortunately, therapeutic attempts to target 4-1BB have been hampered due to hepatotoxicity or suboptimal agonistic potency [16, 43, 44]. In our study, an Fc-muted bispecific antibody, HK010, targeting PD-L1 and 4-1BB was generated and could help T cells relieve the suppression from the PD-1/PD-L1 interaction and stimulate PD-L1-dependent 4-1BB signaling, analogous to “hitting the gas and releasing the brake” described previously [24, 45], thus enhancing T-cell effector function in the TME (see Fig. 8 schematic illustration of the mechanism of action of HK010).

Previously, a 4-1BB agonistic antibody was reported to mediate 4-1BB clustering by binding the Fc domain to FcγR-expressing cells, especially to the inhibitory FcγRIIb [46, 47]. However, this approach was limited due to undesirable toxicity associated with FcγR interactions in the liver and suboptimal efficacy by differential FcγR expression [48]. In addition, FcγRIIb has been described to mediate internalization [49, 50], which may reduce the efficacy of molecules. Some BsAbs targeting 4-1BB and tumor-associated antigens (TAAs), such as human epidermal growth factor receptor 2 (HER2), have the advantages of 4-1BB crosslinking only in the presence of TAAs [51]. Similarly, HK010 was designed as a fully human IgG4-based BsAb with mutations in the Fc region to prevent FcγR binding and thus could mediate 4-1BB clustering by binding to PD-L1 instead of FcγR. Using several reporter gene assays, we confirmed that HK010-mediated 4-1BB costimulation signaling and T-cell activation were strictly dependent on the presence of PD-L1-expressing cells but not FcγR-expressing cells. Meanwhile, HK010 effectively inhibited the PD-1/PD-L1 signaling pathway, indicating that its PD-L1 arm also functions as a classical immune checkpoint inhibitor. Furthermore, it has been demonstrated that a higher affinity for PD-L1 compared with 4-1BB favors the optimal stimulation of T cells [22]. To improve the targeting of tumor cells expressing PD-L1 and reduce possible toxicity, the binding affinity of HK010 to human 4-1BB was designed to be lower than that to human PD-L1 (KD: 493 nM versus 2.27 nM), ensuring a preferential distribution of HK010 to PD-L1-expressing cells in the TME to locally stimulate antigen-specific T cells. Interestingly, HK010 treatment did not injure human PD-L1-expressing monocytes and mDCs, which was in accordance with previous findings [35, 36] and confirmed the safety of HK010.

To understand the binding feature towards antigens of HK010, X-ray crystallography analysis was performed, and the results demonstrated that HK010 interacts with 4-1BB at a unique epitope mainly located at CRD2 of 4-1BB, which is adjacent to the ligand epitope but does not overlap. It was reported that binding to the distal N-terminal domain of 4-1BB should naturally offer a higher degree of agonist activity with potential toxicity [41]. In our study, the 4-1BB ligand-competitive epitope of HK010 mildly activated the downstream signaling pathway when crosslinked with PD-L1. Since PD-L1 is not only a tumor antigen but also a proven immune checkpoint for cancer immunotherapy, the PD-L1 arm of HK010 was screened to block the PD-1/PD-L1 interaction, which was confirmed by crystallography and in vitro bioassays. Through the analysis of the interaction between HK010 and antigens, we proposed a structural model in which HK010 blocks the PD-1/PD-L1 interaction and induces 4-1BB clustering and activation by binding to PD-L1.

HK010 was further confirmed to bind to both antigens simultaneously and bridge PD-L1-/4-1BB-expressing cells using a battery of in vitro functional assays. Moreover, direct evidence from confocal imaging showed that HK010 targeted activated human primary CD8+ T cells to HCC1954 tumor cells and promoted the formation of “immunological synapses” between them, as previously reported [52]. Functionally, HK010 increased the antitumor cytokine release of T cells in a manner strictly dependent on the PD-L1 expression of tumor cells or APCs, thereby facilitating the killing of tumor cells, whereas the anti-4-1BB/PD-L1 antibodies alone or in combination did not exhibit this capacity. In a humanized mouse model bearing human PD-L1-expressing tumors, HK010 displayed superior efficacy in eradicating established tumors even when the dose was as low as 0.3 mg/kg, and could induce durable antigen-specific immune memory to prevent rechallenged tumor growth. Strikingly, HK010 treatment recruited more CD8+ T cells into tumor tissue, reduced TAMs, and resulted in more tumor apoptosis and necrosis than the anti-PD-L1 mAb. These findings demonstrated that HK010 could recruit CD8+ T cells and other cytotoxic lymphocytes into the PD-L1-positive TME and activate TILs by blocking the PD-1/PD-L1 interaction and stimulating the 4-1BB signaling pathway. It was reported that T-cell dysfunction resulting from the immune-suppressive milieu and T-cell exhaustion in the TME leads to immunotherapy resistance in malignancies, including CRC [53, 54]. Therefore, it is possible that HK010 may be used in the therapeutic regimen for ICI-resistant cancer in the future.

It is well known that an antibody Fc can mediate ADCC and CDC [55]. To restrict these off-target effects, HK010 was generated using a human IgG4 backbone with three amino acid mutations (S228P, F234A, L235A) in the Fc region to further minimize the interactions with FcγRs and Fc functional activity, which is supported by the data in our cytotoxicity assay. It was also found that HK010 did not induce nonspecific production of proinflammatory cytokines in vitro. Moreover, according to toxicity studies, HK010 could be well tolerated up to 150 mg/kg with no observed adverse effects in cynomolgus monkeys. These results suggested that HK010 has an excellent safety profile to support further development in clinical studies.

Several important limitations should be considered in our study. First, the antitumor efficacy of HK010 has mainly been studied in CRC, but more cancer types should be tested to explore the indications for HK010 therapy, including those with different expression levels of PD-L1 and resistance to ICI therapy. Second, the mechanisms of HK010 in the formation of antitumor immunity memory, recruitment of lymphocytes, and rescue of exhausted T cells in the TME need to be explored in the future. Currently, HK010 has been approved for evaluation in clinical trials based on preclinical evidence, and multiple types of solid cancers, including CRC, are under investigation.

## Conclusion

We generated a novel anti-PD-L1x4-1BB BsAb, HK010, by fusing an anti-4-1BB scFv at the C-terminus of the heavy chain of an anti-PD-L1 mAb with an Fc-muted mutation and distinguished its structural interactions with PD-L1 and 4-1BB. HK010 exhibits a synergistic effect by blocking the PD-1/PD-L1 signaling pathway and stimulating the 4-1BB signaling pathway simultaneously, and is strictly dependent on the PD-L1 receptor with no systemic toxicity, which may offer the promise of an improved therapeutic window relative to 4-1BB agonists while focusing on the recruitment and stimulation of immune cells in the TME.

## Supplementary Information

**Additional file 1: Table S1.** X-ray diffraction data and refinement statistics. **Table S2.** The expression of PD-L1 in the test cell lines. **Figure S1.** HK010 binds human PD-L1 and 4-1BB simultaneously. **Figure S2.** HK010 has a potent efficacy on MC38/hPD-L1 tumor in a humanized mouse model. **Figure S3.** HK010 shows antitumor activity strictly on human PD-L1-expressing tumor. **Figure S4.** HK010 exhibits cross-reactivity between human and cynomolgus monkey.

## Data Availability

The data supporting the findings of this study are available within the article and its Additional file 1 and from the corresponding authors upon reasonable request. Source data are provided with this paper.

## Abbreviations

ADCC: Antibody-dependent cell-mediated cytotoxicity
AUC: Area under the curve
BsAb: Bispecific antibody
CDC: Complement-dependent cytotoxicity
CFSE: Carboxyfluorescein succinimidyl ester
CRC: Colorectal cancer
CRD: Cysteine-rich domain
iDCs: Immature dendritic cells
mDCs: Mature dendritic cells
ECD: Extracellular domain
EC50: Half-maximal effective dose
ELISA: Enzyme-linked immunosorbent assay
Fab: Antigen-binding fragment
Fc: Crystallizable fragment
HE: Hematoxylin and eosin
mAb: Monoclonal antibody
mIHC: Multiplexed immunohistochemistry
MLR: Mixed lymphocyte reaction
MTD: Maximum tolerated dose
NK: Natural killer cell
PBMC: Peripheral blood mononuclear cell
PBST: Phosphate buffered solution containing 0.05% Tween-20
PD-1: Programmed cell death-1
PD-L1: Programmed cell death ligand 1
PK: Pharmacokinetics
scFv: Single-chain variable fragment
SPR: Surface plasmon resonance
TCR: T-cell receptor
TILs: Tumor-infiltrating lymphocytes
TMB: Tetramethylbenzidine
TME: Tumor microenvironment
TNFR: Tumor necrosis factor receptor
