# Supplementary material for: An Fc-muted bispecific antibody targeting PD-L1 and 4-1BB induces antitumor immune activity in colorectal cancer without systemic toxicity
Source: Cell Mol Biol Lett. 2023 May 31;28:47. doi: 10.1186/s11658-023-00461-w (PMC10230818; doi:10.1186/s11658-023-00461-w)
Supplement: Supplementary file 1 — Additional file 1: Table S1. X-ray diffraction data and refinement statistics. Table S2. The expression of PD-L1 in the test cell lines. Figure S1. HK010 binds human PD-L1 and 4-1BB simultaneously. Figure S2. HK010 has a potent efficacy on MC38/hPD-L1 tumor in a humanized mouse model. Figure S3. HK010 shows antitumor activity strictly on human PD-L1-expressing tumor. Figure S4. HK010 exhibits cross-reactivity between human and cynomolgus monkey. [file 11658_2023_461_MOESM1_ESM.docx]

**Additional Table S1 X-ray diffraction data and refinement statistics**

| **Data set** | **HuB6 Fab/4-1BB** | **HK010 Fab/PD-L1** |
| --- | --- | --- |
| Wavelength (Å) | 0.97915 | 1.54056 |
| Space group | *P*1 | *P*2_1_2_1_2_1_ |
| Unit cell (Å,°) | *a*=60.021 *b*=65.913 *c*=83.643 | *a*=71.08 *b*=94.88 *c*=96.07 |
|  | *α*=90 *β*=82.495 *γ*=74.822 | *α*=*β*=*γ*=90 |
| Resolution range (Å) | 19.91-2.30(2.37-2.30) | 13.14-2.30(2.382-2.30) |
| Unique reflections | 52988(5290) | 29324(2885) |
| Completeness (%) | 97.19(97.17) | 99.27(99.55) |
| Redundancy | 3.5(3.5) | 2.0(2.0) |
| Average (I/sigma) | 7.8(1.5) | 8.2(1.3) |
| *R*_merge_ | 0.085(0.821) | 0.109(0.582) |
| CC (1/2) | 0.995(0.785) | 0.983(0.455) |
| Wilson B-factor (Å^2^) | 46.08 | 24.45 |
| Solvent content (%) | 49.46 | 51.67 |
| No.of subunits per asymmetric unit | 2 | 1 |
| *R*_factor_% | 22.2 | 19.43 |
| *R*_free_% | 27.7 | 24.62 |
| R.m.s.d.bond length (Å) | 0.004 | 0.004 |
| R.m.s.d.bond angle (°) | 0.62 | 0.99 |
| No. of water molecules | 128 | 398 |
| *B*-factor (Å^2^) |  |  |
| Macromolecules | 62.64 | 29.67 |
| Ligands | 87.02 | None |
| Solvent | 56.3 | 31.31 |
| Ramachandran plot (%) |  |  |
| Residues in most favored regions | 98.22 | 97.0 |
| Residues in allowed regions | 1.78 | 2.8 |
| Residues in outlier regions | 0 | 0.2 |

**Additional Table S2 The expression of PD-L1 in the test cell lines**

| **Cell line** | **Blank MFI** | **Detection MFI** | **Expression MFI** |
| --- | --- | --- | --- |
| HCC1954 | 4126.9 | 142366.3 | 138239.4 |
| MDA-MB-231 | 1113 | 22733 | 21620 |
| HCC827 | 2339.5 | 30267.6 | 27928.1 |
| HT29 | 680.8 | 2029.3 | 1348.5 |
| CHO-K1 | 381.8 | 423.4 | 41.6 |

MFI, mean fluorescence intensity.


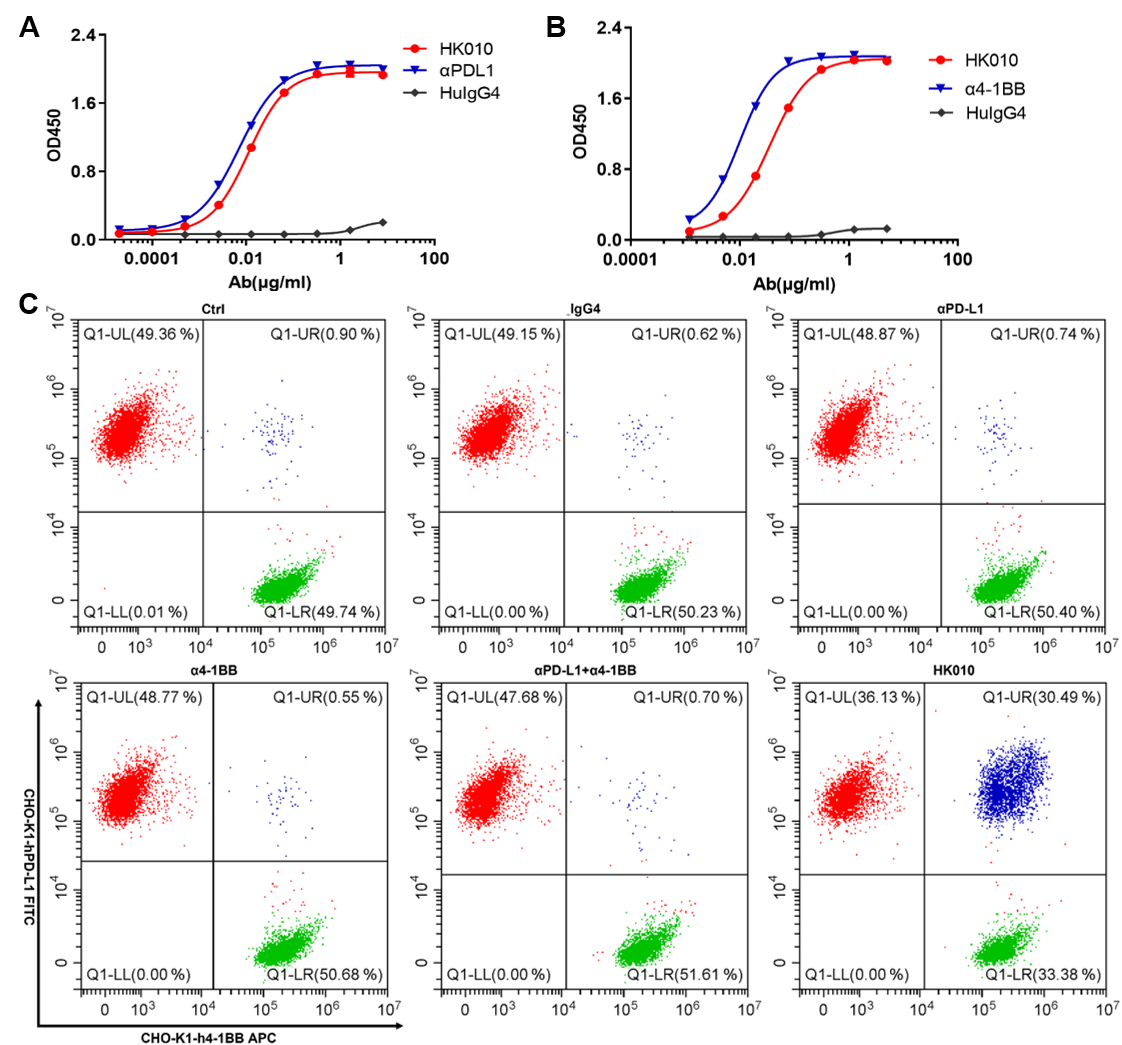


**Additional Fig. S1 HK010 binds human PD-L1 and 4-1BB simultaneously.** (A) HK010 binds to human PD-L1, as shown by ELISA. (B) HK010 binds to human 4-1BB, as shown by ELISA. (C) HK010 binds to CHO-K1-hPD-L1 cells with human PD-L1 expression and CHO-K1-h4-1BB cells with human 4-1BB expression, as shown by flow cytometry. One representative result from three independent experiments is shown.


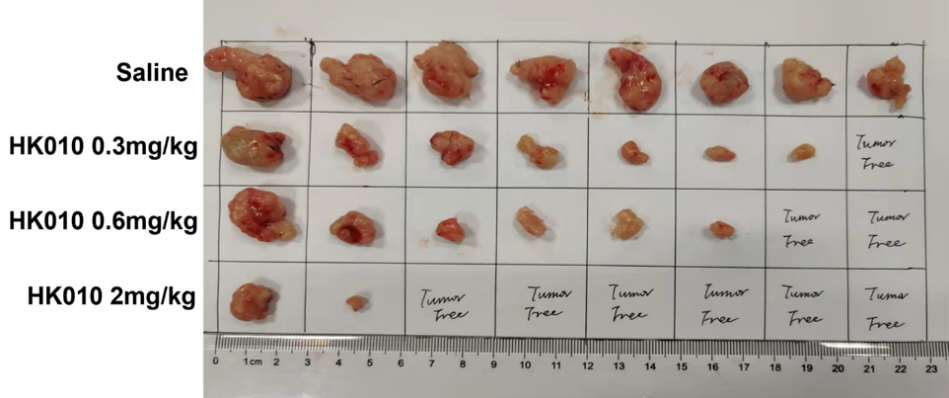


**Additional Fig. S2** **HK010 has a potent efficacy on MC38/hPD-L1 tumor in a humanized mouse model.**

**
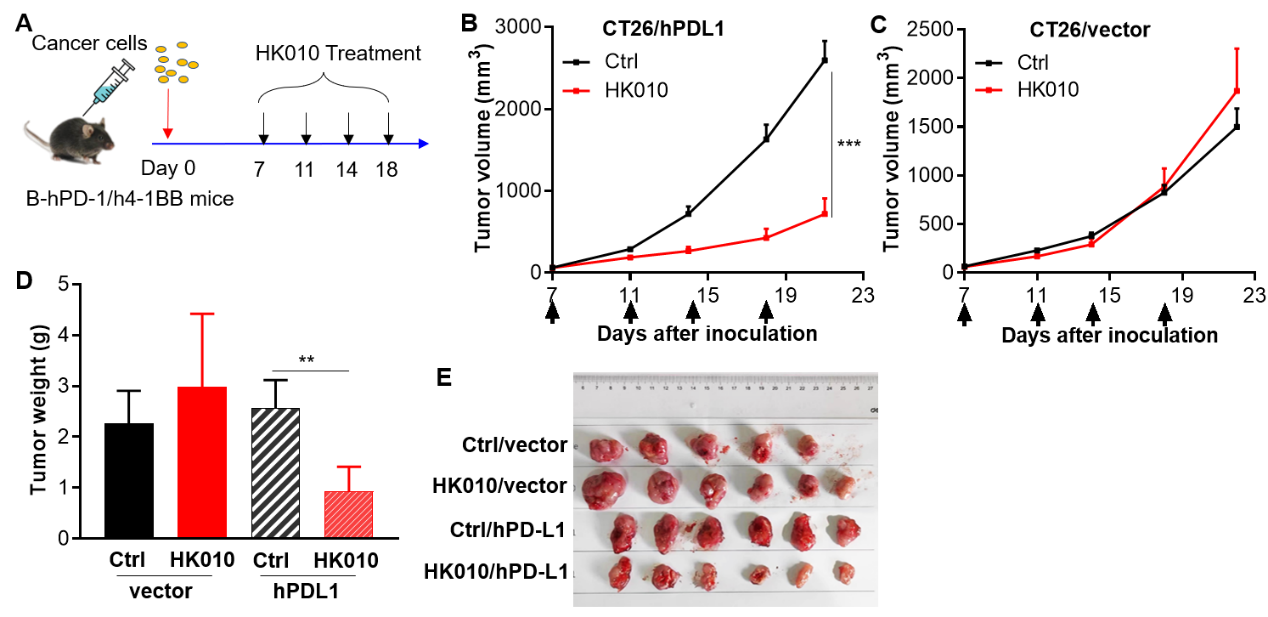
**

**Additional Fig. S3 HK010 shows antitumor activity strictly on** **human PD-L1-expressing tumor.** (A) Schematic diagram of HK010 treatment of B-hPD-1/h4-1BB mice bearing CT26/hPD-L1 or CT26/vector transplants. When the mean tumor size reached approximately 70 mm^3^, mice were randomized into groups of 6 animals per group. Treatment with HK010 four times (indicated by vertical arrows). The changes in tumor volume (B, C) and tumor weight (D) of the mice treated with HK010. The values are presented as the mean ± SD from one representative of three independent experiments. **p < 0.01, ***p < 0.001. (E) Photo of HK010 efficacy on CT26/hPD-L1 and CT26/vector tumors in a humanized mouse model.


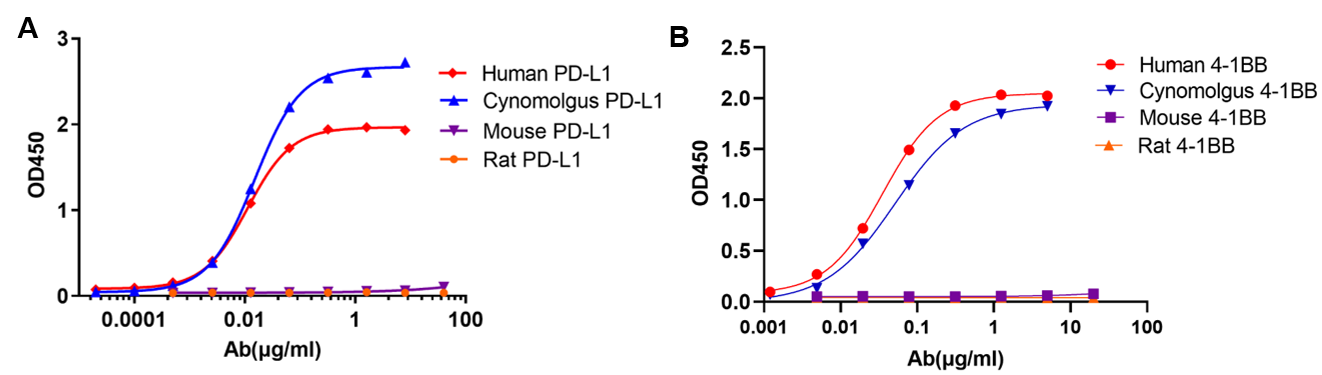


**Additional Fig. S4 HK010 exhibits cross-reactivity between human and cynomolgus monkey.** (A) The cross-reactivity with PD-L1 of different species was determined by ELISA. (B) The cross-reactivity of 4-1BB with PD-L1 from different species was determined by ELISA.
